# Targeting Bcl-2/Bcl-X_L_ Induces Antitumor Activity in Uveal Melanoma Patient-Derived Xenografts

**DOI:** 10.1371/journal.pone.0080836

**Published:** 2014-01-13

**Authors:** Fariba Némati, Catherine de Montrion, Guillaume Lang, Laurence Kraus-Berthier, Guillaume Carita, Xavier Sastre-Garau, Aurélie Berniard, David Vallerand, Olivier Geneste, Ludmilla de Plater, Alain Pierré, Brian Lockhart, Laurence Desjardins, Sophie Piperno-Neumann, Stéphane Depil, Didier Decaudin

**Affiliations:** 1 Laboratory of Preclinical Investigation, Department of Translational Research, Institut Curie, Paris, France; 2 I.D.R.S., Institut de Recherches Servier, Croissy, France; 3 I.R.I.S., Institut de Recherches International Servier, Suresnes, France; 4 Department of Tumor Biology, Institut Curie, Paris, France; 5 Department of Ophthalmological Oncology, Institut Curie, Paris, France; 6 Department of Medical Oncology, Institut Curie, Paris, France; The Moffitt Cancer Center & Research Institute, United States of America

## Abstract

**Purpose:**

Uveal melanoma (UM) is associated with a high risk of metastases and lack of efficient therapies. Reduced capacity for apoptosis induction by chemotherapies is one obstacle to efficient treatments. Human UM is characterized by high expression of the anti-apoptotic protein Bcl-2. Consequently, regulators of apoptosis such as Bcl-2 family inhibitors may constitute an attractive approach to UM therapeutics. In this aim, we have investigated the efficacy of the Bcl-2/Bcl-X_L_ inhibitor S44563 on 4 UM Patient-Derived Xenografts (PDXs) and derived-cell lines.

**Experimental Design:**

Four well characterized UM PDXs were used for *in vivo* experiments. S44563 was administered alone or combined with fotemustine either concomitantly or after the alkylating agent. Bcl-2, Bcl-X_L_, and Mcl-1 expressions after S44563 administration were evaluated by immunohistochemistry (IHC).

**Results:**

S44563 administered alone by at 50 and 100 mg/kg i.p. induced a significant tumour growth inhibition in only one xenograft model with a clear dose effect. However, when S44563 was concomitantly administered with fotemustine, we observed a synergistic activity in 3 out of the 4 tested models. In addition, S44563 administered after fotemustine induced a tumour growth delay in 2 out of 3 tested xenografts. Finally, IHC analyses showed that Bcl-2, Bcl-X_L_, and Mcl-1 expression were not modified after S44563 administration.

**Conclusion:**

The novel anti-apoptotic experimental compound S44563, despite a relative low efficacy when administered alone, increased the efficacy of fotemustine in either concomitant or sequential combinations or indeed subsequent to fotemustine. These data support further exploration of potential therapeutic effect of Bcl-2/Bcl-xl inhibition in human UM.

## Introduction

Uveal melanomas (UM) represent the most frequent intraocular tumour in adult patients. Whereas the 5-year overall survival rate of localized disease is greater than 70%, the prognosis drops dramatically in cases of metastases [1]. Up to 50% of patients will develop metastases within a median time of 2.4 years [2]. The overall survival is poor, and the majority of patients will succumb to their cancer. Systemic therapy with alkylating agents, i.e. fotemustine, dacarbazine, or temozolomide, have shown only modest efficacy [3]. Consequently, because of the limited efficacy of current treatments, new therapeutic strategies need to be developed.

One of the primary means by which UM cells evade treatment-induced apoptosis is by up-regulation of members of the prosurvival Bcl-2 family proteins such as Bcl-2 and Bcl-XL [4], [5]. Indeed, human uveal melanomas are characterized by a high average percentage of Bcl-2 positive cells of 82% (range: 44%–100%) [6]–[12], but without any prognostic impact [6], [9], [10], [12]. This observation was also confirmed by the present group in a panel of primary human UM xenografts obtained from patient's tumors, where Bcl-2 was shown to be expressed in almost all studied patient's tumours and corresponding xenografts [13].

A variety of approaches to target these anti-apoptotic oncoproteins have been pursued in order to try and restore the natural process of programmed cell death [14], notably bcl-2 anti-sense oligonucleotides such as Oblimersen (Genasense®) with contrasted positive [15], [16] or negative [17]–[19] impact in randomized clinical trials performed in cutaneous melanoma, chronic lymphoid leukemia, multiple myeloma, and prostate cancer patients. Another potential therapeutic approach consists of using small molecules that occupy the BH3 binding groove of antiapoptotic Bcl-2 family members (BH3 mimetics), including ABT-737 [20], ABT-263 (Navitoclax®) [21], and GX15-070 (Obatoclax®) [22]. These drugs disrupt Bcl-2/Bcl-X_L_ interactions with pro-death proteins (e.g., Bim), leading to the initiation of apoptosis. In human tumor cells, BH3 mimetics induce Bax translocation, cytochrome c release, and subsequent apoptosis. In human xenograft models of small-cell lung cancer, multiple myeloma, lymphoblastic leukaemia, and aggressive B-cell lymphoma, Bcl-2/Bcl-X_L_ inhibitors were previously shown to significantly enhance the efficacy of clinically relevant therapeutic regimens [21], [23]–[25]. As relatively few clinical studies using these new compounds have been reported [26], [27], no randomized clinical data are already available.

In contrast to the high number of reports in various hematologic and solid tumors including cutaneous melanoma, only one *in vitro* study has evaluated Bcl-2 targeting in uveal melanoma cells, showing synergistic effect with chemotherapy and multi-drug resistance reversion [28]. Based on this observation and considering the high Bcl-2 expression in UM and the occurrence of liver metastases in its natural history, we have evaluated the therapeutic potential of a new experimental Bcl-2/Bcl-X_L_ inhibitor, S44563, in different uveal melanoma Patient-Derived Xenografts (PDXs), and derived-cell lines, alone or in combination with fotemustine.

## Materials and Methods

### Ethics statement

Before PDX establishment, all patients had previously given their verbal informed consent for experimental research on residual tumor tissue available after histophatologic and cytogenetic analyses. Those PDXs establishments have been performed after approval of the ethics committee of the Institut Curie. According to the French rules and the ethics committee of the Institut Curie, a written consent from patients for obtaining residual tumor tissues is not required. In case of patient refusal that could be orally expressed or written, residual tumor tissues are not collected. All issues that patients want to be discussed could be raised at any time during any medical consultations. This procedure was approved by ethics committees. This research was not conducted outside of our country. Studies have been performed in compliance with the recommendations of the French Ethical Committee and under the supervision of authorized investigators. The experimental protocol and animal housing were in accordance with institutional guidelines as put forth by the French Ethical Committee (Agreement C75-05 - 18, France), and the ethics committee of the Institut Curie that approved this project.

### Compounds

S44563 is a new Bcl-2/bcl-X_L_ inhibitor. It potentially inhibits the anti-apoptotic proteins Bcl-2 and Bcl-X_L_ by binding to their respective BH3 binding groove. S44563 was synthesized at IdRS (France) as described (initial patent application filed in France 02/02/2007 under N°07/00741). S44563 (C_44_H_47_CIN_6_0_5_S_2_) has a molecular weight of 912.4 (chemical structure is presented in Figure S1). S44563-2 was synthesized at Institut de Recherches Servier. The purity of the batches ranges between 97.8% and 99.9%, and the optical purity was 99%. Fotemustine (Muphoran®) was kindly provided from IRIS (France).

### Fluorescence polarisation assays

A fluorescent BH3 peptide (BH3 motif from the protein Puma) was incubated with either recombinant Bcl-2 or Bcl-X_L_ protein (20 mM Na_2_HPO_4_ pH 7.4, 50 mM NaCl, 1 mM EDTA, 0.05% pluronic acid). Upon binding, the fluorescence emitted by the BH3 peptide becomes polarised. The inhibition of the interaction between the fluorescent BH3 peptide and Bcl-2 or Bcl-X_L_ is followed by measuring a decrease in fluorescence polarisation. In these assays, Bcl-2 or Bcl-X_L_ concentration was 100 nM, the fluorescent BH3 peptide concentration was 15 nM and S44563-2 was titrated from 1 nM to 100 µM.

### Uveal melanoma PDXs

Three UM cell lines have been used, all derived from UM PDXs established from intraocular lesion (MP41, MM26, and MM66). Four UM PDXs obtained from patients after enucleation (MP41 and MP77) or liver metastasis surgery (MM26 and MM66), established, characterized and maintained into SCID mice have been used [13], [29]. The principal characteristics of these 4 xenografts are presented in the Table S1. We could not use each model in both *in vitro* and *in vivo* experiments, because some xenografts could not be maintained in prolonged *in vitro* cultures.

### In vitro experiments

UM cell lines were routinely maintained in RPMI 1640 containing GlutaMAX™ (Invitrogen, Carlsbad, CA, USA), 20% fetal bovine serum (Invitrogen, Carlsbad, CA, USA) and 1% penicillin and streptomycin (Invitrogen, Carlsbad, CA, USA). Flasks of 75 cm^3^ were incubated at 37°C in humidified 5% CO_2_ until 85%–95% confluence was attained. Thereafter, cells were seeded (2×10^5^ cells/ml) in 96-well plates, incubated 24 h at 37°C, and then treated with different doses of S44563 (dissolved at 20% DMSO and diluted with 0.9% sodium chloride at 20 mg/ml) (Servier, Courbevoie, France). The cells were incubated for 24 h at 37°C.

Cell viability was determined with WST-1 assay (Roche, Indianapolis, USA). The culture medium was replaced with a medium containing a WST-1 reagent, and 1 h later, the absorbance in the well was determined at 450 nm with a reference wavelength of 620 nm by using a microplate reader TECAN Infinite M200 (TECAN Deutschland GmbH, Crailsheim, Germany, Software Magellan™). The percentage of viability was evaluated by ΔOD_(540–620)_ treated/ΔOD_(540–620)_ control×100. The 50% growth inhibitory concentration (IC_50_) (50% inhibitory concentration) was then defined for each drug and cell line.

For determination of apoptosis, mitochondrial transmembrane potential (ΔΨm) and cell cycle, cells were incubated with S44563 at a concentration of 17 and 34 µM for 24 h. The detection of apoptosis and ΔΨm alterations was done by the protocol AbC Annexin 5/FITC kit (AbCys, Paris, France) and BD™ MitoScreen kit (BD Biosciences, San Jose, CA, USA) respectively with DAPI (1/200, Invitrogen, Carlsbad, CA, USA). The study of the cell cycle by propidium iodure (1/100, Sigma-Aldrich Chemie, Saint-Quentin Fallavier, France) was performed after fixing cells with ethanol 70% and permeabilization with RNase A (1/100, Roche, Indianapolis, IN).

Cell autophagy was assessed by detecting cleaved and non-cleaved LC3 [30], cells were seeded and treated by 2 and 4 µM of S44563 in 6-well plates for 24 hours. Cell pellets were lysed in 30 µl of boiling Laemmli lysis buffer [50 mM Tris pH = 6.8, 2% SDS, 5% glycerol, 2 mM DTT, 2.5 mM EDTA, 2.5 mM EGTA, qsp H_2_O, Phosphatase inhibitor 1× (Halt Phosphatase inhibitor cocktail, Perbio Science France, Brebières, France), protease inhibitors 1 tablet/10 ml (Protease inhibitor cocktail, complete MINI EDTA-free, Roche, Indianapolis, IN), 2 mM Orthovanadate of Sodium and 10 mM Sodium Fluoride]. Total proteins were determined by BCA Protein assay kit reducing Agent compatible (Perbio Science France, Brebières, France). Total proteins were separated by Invitrogen gel 12% (Buffer NuPAGE MES 1× and anti-oxydant, Invitrogen, Carlsbad, CA, USA), transferred to nitrocellulose membranes and analyzed by immunoblotting using the ECL method (ECL Western Blotting Detection Reagents, GE Healthcare Life Sciences GmbH, Velizy-Villacoublay, France). The following primary antibodies were used: anti-LC3B antibody produced in rabbit (1/500, Sigma-Aldrich Chemie, Saint-Quentin Fallavier, France), anti-β-actin antibody produced in mouse (1/5000, Sigma-Aldrich Chemie, Saint-Quentin Fallavier, France). Polyclonal goat anti-rabbit (1/5000) and anti-mouse (1/10000) secondary antibody conjugated horseradish peroxidase (HRP) were purchased from Dako France (Trappes, France) and Jackson ImmunoResearch Laboratories (West Grove, PA, USA) respectively. The signals were detected by LAS-3000 imager (Fujifilm, Odawara, Japan). Membranes were exposed during 5 minutes. The quantity of cleaved (active) and inactive (non-cleaved) LC3 was related to ß actin.

All results were statistically analyzed by two-way ANOVA test with Bonferroni post-test *versus* control group. Data were represented as mean ± standard error of mean (n = 3–5 replicates). p<0.05 were considered significant (*).

### In vivo experiments

SCID mice, 4 to 6 weeks old, bred at Curie Institute, were used for *in vivo* experiments. Fragments of 30–60 mm^3^ were grafted subcutaneously into the interscapular fat pad. When tumors reached a size of 60–200 mm^3^, mice were randomly assigned to the control or treatment groups and treatment was started on day 1. Between 8 to 12 mice per group were included in *in vivo* experiments. Tumor growth was evaluated by measurement of two perpendicular diameters of tumors with a caliper, twice a week. Individual tumor volume, relative tumor volume (RTV), and tumor growth inhibition (TGI) were calculated according to standard methodology [13]. Statistical significance of TGI was calculated by the paired Student's t-test. Xenografted mice were sacrificed when their tumor reached a volume of 2500 mm^3^.

For *in vivo* administration, S44563 was diluted in polysorbate 80/water 10%.and was administered at a dose of 50 or 100 mg/kg alone, concomitantly (days 1–5/8–12/22–26/29–33) and/or after (days 43–47/50–54/64–68/70–74) fotemustine administration (15 or 30 mg/kg days 1 and 22). All treatments were administered intraperitoneally.

In all *in vivo* experiments, a relative tumor volume variation (RTVV) of each S44563-treated mouse was calculated from the following formula: [(Vt/Vc)−1], where Vt is the volume of the treated mouse and Vc the median volume of the corresponding control group at a time corresponding to the end of treatment.

### Immunohistochemical studies

All immunohistochemistry protocols were realized automatically on a Ventana™ Discovery XT machine with neutral buffered formalin-fixed (4%) tissue sections of paraffin-embedded tumor biopsies. In each case, 5-µm sections were pre-treated with standard CC1 reagent (Ventana™ Tris/Borate/EDTA buffer pH 8- 48 min- 95°C) for Bcl-2,) or mild CC1 (28 min) for Mcl-1 and with standard CC2 reagent (Ventana™ Citrate buffer pH 6 48 min 95°C) for Bcl-XL, cleaved Caspase 3, and LC3B. Endogenous biotin blocking was carried out for all antibodies detections (8 min). The following primary antibodies were used: rabbit monoclonal anti-human Bcl-2 (clone E17) from Epitomics (ref 1017-1) dilution 1∶100, 60 min incubation; rabbit monoclonal anti-human Bcl-X_L_ (clone 54H6) from Cell Signaling Technology (ref 2764) dilution 1∶150, 60 min incubation; rabbit polyclonal anti-human Mcl-1 from Sigma (ref HPA008455 dilution 1∶80, 60 min incubation; rabbit monoclonal anti-human cleaved caspase 3 (clone C92-605) from Pharmingen (ref 559565) dilution 1∶100, 32 min incubation; rabbit monoclonal anti LC3B (clone D11) from Cell Signaling Technology (ref 3868) dilution 1∶100 60 min incubation. Biotinylated donkey anti rabbit IgG from Jackson Immunoresearch (ref 711-065-152) was added in sequence (dilution 1∶100 32 min incubation. Excepted for Caspase 3 and LC3B detections on MM26 and MP41, a chromogen substrate (DAB diaminobenzidine-biotin-peroxidase) was added to each specimen according the manufacter's instructions. Finally, sections were counter-stained (hematoxylin: 8 min) and post counter-stained (bluing reagent: 8 min). For caspase 3 and LC3B detections on MM26 and MP41 tumours the chromogenic system was NBT/BCIP and sections were counter-stained with Nuclear Fast Red. All the incubations were performed at 37°C with the exception of pre-treatments (95°C).

Intensity and percentage of positive stained (DAB) cells were then quantified by an anatomopathologist (Dr J.F. Boivin). Two parameters were used to evaluate Bcl-2, Bcl-X_L_, and Mcl-1 expression: semi-quantitative visual grading system of the intensity of staining (−, negative; +, weak; ++ modest; +++ strong), and the percentage of labelled tumor cells. To integrate these two parameters, a H-score (histological score) was calculated as H-score = tumor cell staining percentage×staining intensity. One parameter (percentage of labeled tumor cells) was used to evaluate cleaved Caspase 3.

## Results

### Inhibition of Bcl-2 and Bcl-X_L_ binding to a BH3 peptide by S44563

**Figure 1:**
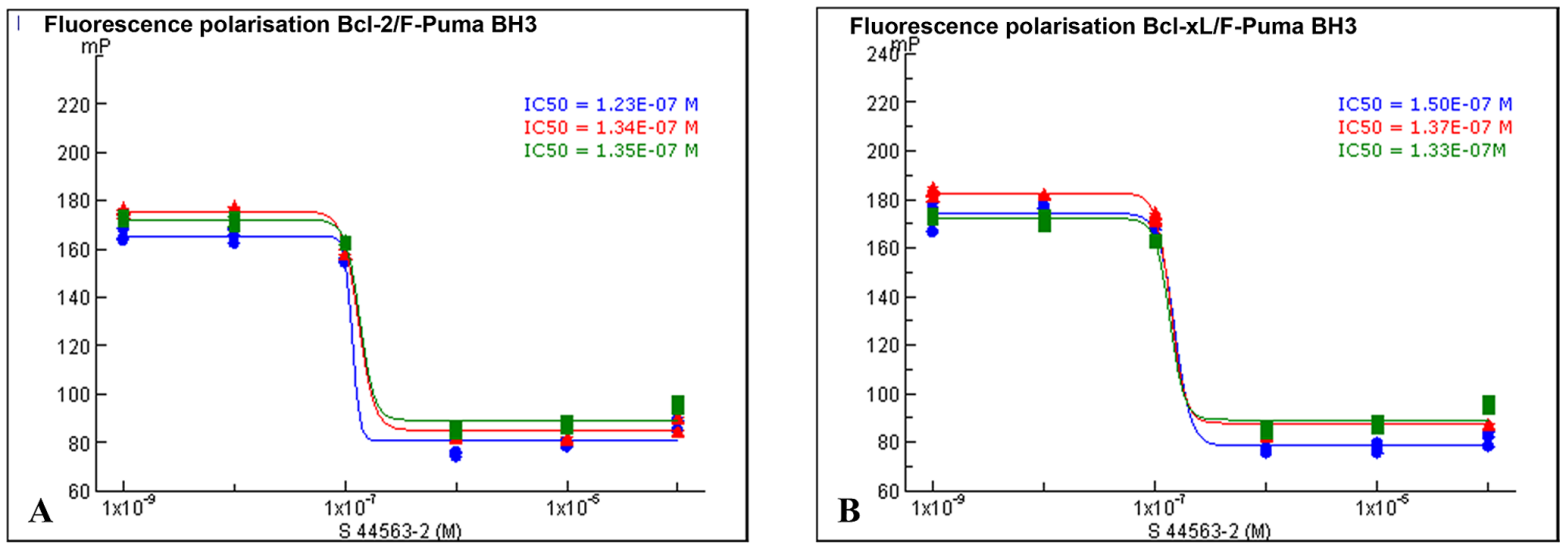
Inhibition of the interaction between Bcl-2 or Bcl-X_L_ and fluorescent Puma BH3 peptide measured by the decrease of fluorescence polarisation as a function of S44563-2 concentration. Three independent experiments are presented. FP data are presented in millipolarization units (mP). For each experiment, measures are assessed in triplicate.

Inhibition of Bcl-2 and Bcl-X_L_ was investigated by fluorescence polarisation (FP) assays using recombinant Bcl-2 or Bcl-X_L_ and a fluorescent Puma BH3 peptide. The Figures 1A and 1B show the inhibition of Bcl-2 and Bcl-X_L_ respectively by S44563. S44563 is a potent inhibitor of Bcl-2 and Bcl-X_L_. Its IC_50s_ in a Bcl-2/F-PumaBH3 interaction assay and a Bcl-X_L_/F-PumaBH3 interaction assay were 131 nM [123 nM;135 nM] and 140 nM [133 nM;150 nM], respectively.

### Bcl-2, Bcl-X_L_, and Mcl-1 expression in uveal melanoma PDXs

**Figure 2:**
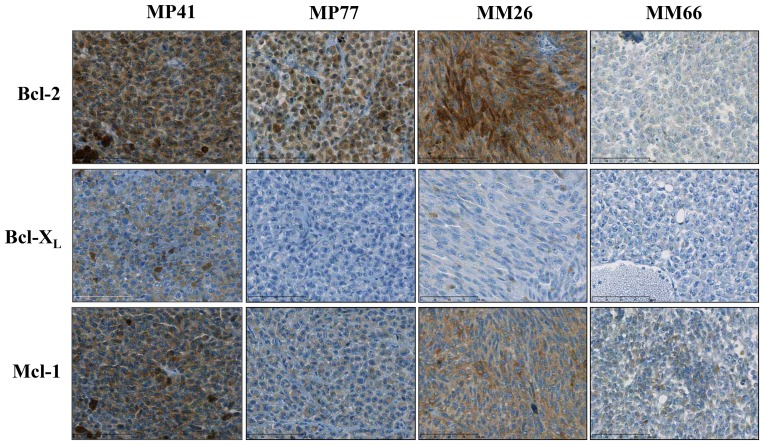
Bcl-2, Bcl-X_L_, and Mcl-1 Immunohistochemistry. Bcl-2, Bcl-X_L_, and Mcl-1 expression determined by immunohistochemical analyses of the 4 human UM xenografts (between 3 to 5 tumors have been studied per condition).

The determination of the expression of Bcl-2, Bcl-X_L_, and Mcl-1 was performed in 3 to 5 different tumors for each model. The expression of Bcl-2 was high for 3 UM xenografts used for *in vivo* experiments (MP41, MP77, and MM26), where the median H-score ranged between 150 and 175, and low for the MM66 model for which the median Bcl-2 H-score was lower than 5 (Table S2) (Figure 2). In contrast, Bcl-X_L_ was highly expressed in the MP41 xenograft (H-score of 175) and not or slightly expressed in the 3 remaining models (Table S1) (Figure 2). Finally, Mcl-1 was expressed in a high proportion of tumor cells of all studied xenografts, except for the MM66 xenograft, but with a relative weak intensity (Table S1) (Figure 2). A global IHC score was then defined, including Bcl-2, Bcl-X_L_, and Mcl-1 protein expression, as: (Bcl-2 score)+(Bcl-X_L_ score)/Mcl-1 score. The median global score differed among the 4 studied models, i.e. 3.25 for the MP41 xenograft, 1.19 for the MP77 model, 2.06 for the MM26 xenograft, and 0.33 for the MM66 model.

### In vitro studies

**Figure 3:**
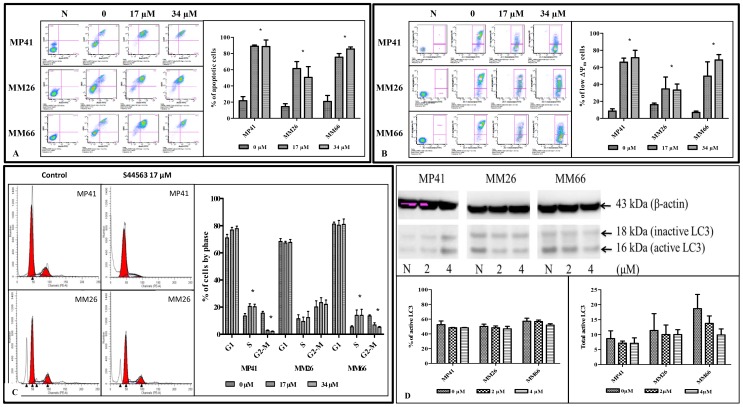
*In vitro* apoptosis induction by S44563. A and B. Apoptosis induction by S44563. The MP41, MM26, and MM66 cell lines were incubated with 17 µM and 34 µM S44563 for 24 hours, after which the samples were labeled with DAPI/annexin V-FITC (A) or JC1 (B). The proportion of annexin V-positive cells and low Δψ_m_ cells was indicated in C and E, respectively. A two-way ANOVA with Bonferroni post-test was then performed (* p<0.05). **C.** Cell cycle analyses after S44563 exposure (17 or 34 µM for 24 h) in the 3 UM cell lines. Cell cycle analysis was determined by labeling the DNA with propidium iodide. Each of the three lines was treated by 17 µM or 34 µM of S44563. The proportion of cells (%) in different cell cycle phases was compared with the control (untreated). Two-way ANOVA with Bonferroni post-test was then performed (* means a p<0.05). **D.** Cell cycle analyses of the xMP41 UM cell line. I and J. Measurement of autophagy on the 3 UM cell lines after S44563 exposure (2 or 4 µM for 24 h). After S44563 treatment, the amount of active and inactive protein LC3 was determined and reported to ß-actine. The quantity of total LC3 protein was calculated to study the activation of LC3. Results were presented as percentage and total amount of active cleaved LC3, relative to β-actin. A two-way ANOVA with Bonferroni post-test was then performed (* means a p<0.05).

For the 3 uveal melanoma xenograft-derived cell lines MP41, MM26, and MM66 incubated with increased concentrations of S44563, 17 µM and 34 µM for 24 hours, the IC50s were 4 µM, 7 µM, and 6 µM for the MP41, MM26, and MM66 cell lines, respectively (Figure S2). Similarly, to evaluate apoptosis induction, the MP41, MM26, and MM66 cell lines were incubated with 17 µM and 34 µM S44563 for 24 hours, after which the samples were labeled with DAPI/annexin V-FITC or JC1. We observed apoptosis induction and a drop of the ΔΨm in the 3 UM cell lines, although the effects were less important in the MM26 cell line (Figure 3A and 3B). Similarly, in the two UM cell lines MP41 and MM66 that are more sensitive to S44563, we observed a significant decrease of the G2/M phases which were not modified in the less sensitive MM26 cell line (Figure 3C).

To study the impact on autophagy induction of S44563, the 3 UM cell lines were cultured with non-apoptotic doses of S44563 (2 and 4 µM for 24 hours). The amount of active (cleaved form) and inactive (non-cleaved form) protein LC3 was measured by western blotting. We observed that both the proportion and the total amount of cleaved LC3 protein relative to β-actin, were not significantly modified after S44563 administration in the 3 studied UM cell lines (Figure 3D).

### In vivo experiments

**Figure 4:**
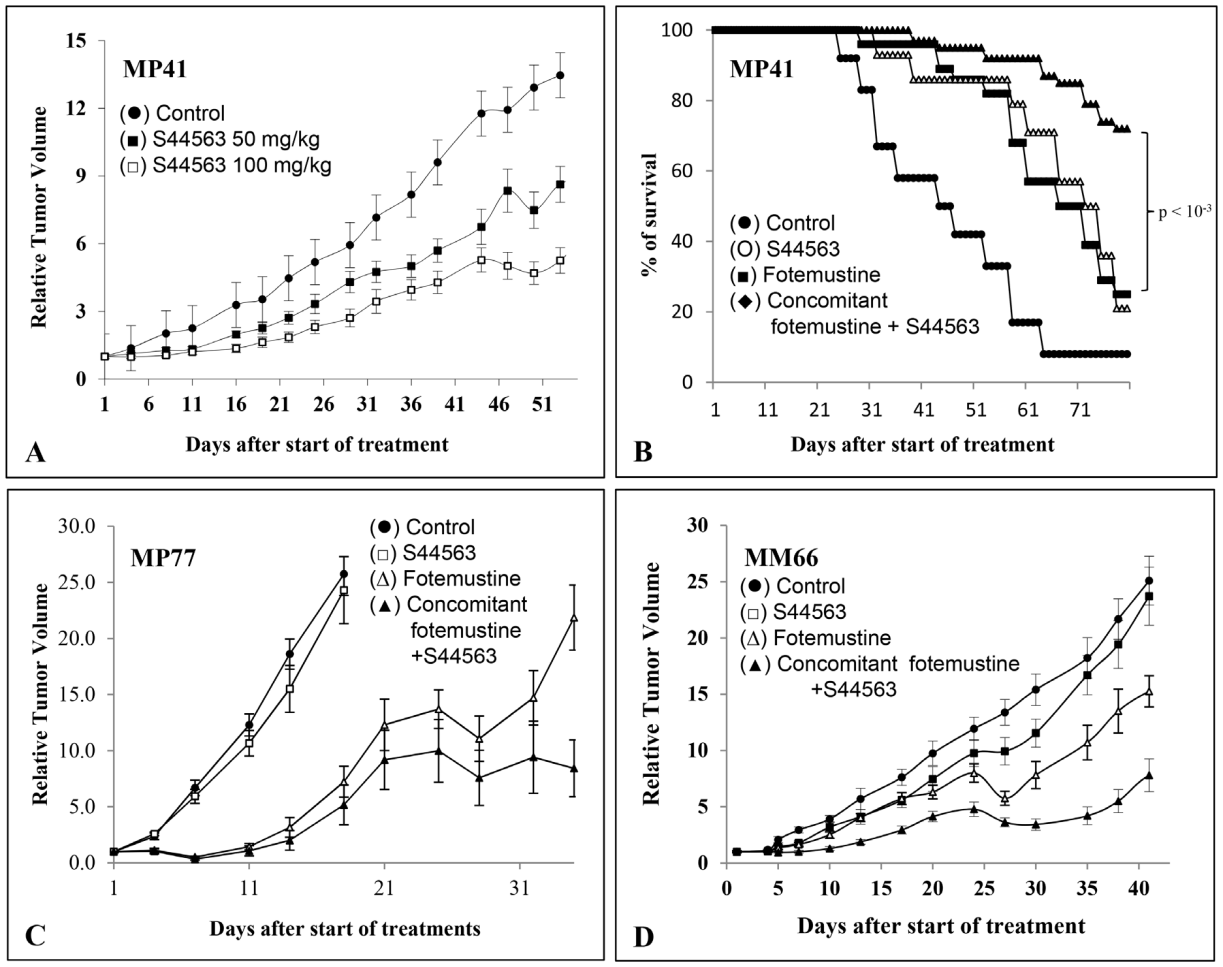
*In vivo* responses of UM PDXs to S44563 administered alone or in combination with fotemustine. A. MP41 xenograft was treated either by S44563 at 50/kg (▪) or 100 mg/kg (□) 5 days a week for 5 weeks. **B.** Overall survival of mice bearing MP41 tumors treated by S44563 (O), fotemustine (▪), and concomitant fotemustine+S44563 (⧫). **C.** MP77 xenograft was treated by S44563 at 100 mg/kg (□), fotemustine 15 mg/kg (Δ), or both (▴). **D.** MM66 xenograft was treated by S44563 at 50 mg/kg (□), fotemustine 30 mg/kg (Δ), or both (▴). Mice in the control group (•) received 0,3 ml of the drug-formulating vehicle with the same schedule as the treated animals. Tumor growth was evaluated by plotting the mean of the RTV (relative tumor volume) ± SD per group. Between 8 to 12 mice per group were included in *in vivo* experiments.

In the 4 UM PDXs, S44563 administered alone at 50 and 100 mg/kg, for 2 cycles of treatment (5 days a week for 2 weeks and 1 week off, each cycle), induced a significant antitumor effect in one model (MP41), with a dose-dependent response and an optimal TGI of 61% at day 53 after start of treatment (Table 1) (Figure 4A). To evaluate responses to S44563 observed in the 4 models according to individual mouse variability, we decided to consider each mouse as one tumor-bearing entity. In this, when relative tumor volume variation [(RTVV)−1] of each S44563-treated mouse was calculated, regardless of the doses of S44563, we observed that 68% of all S44563-treated mice (59/87) had a negative ratio compared to control groups, and that 22% had a ratio lower than 50%, which constitute the threshold to consider a model as responding. Moreover, the overall response rate (RTVV≤50%) was 11% for the 50 mg/kg per injection of S44563, and 32% for the 100 mg/kg dose (p<0.05), confirming the dose-dependent efficacy of the Bcl-2/Bcl-X_L_ inhibitor (Figures 3A and S3B).

**Table 1:** TGI and complete remission induced by a combination of S44563 and fotemustine.

Treatments	MP41	MP77	MM26	MM66
S-50	36	0	0	0
S-100	61	12	10	6
**F-15**	**/**	**72**	**/**	**19**
**F-15+S-100**	**/**	**80**	**/**	**40**
F-30	64	95 (8/10)	84 (0)	39
F-30+S-50	73	99 (8/10)	97 (4/9)	69*

Complete remissions are indicated in brackets.

p<0.005.

We then evaluated administration of both S44563 and fotemustine (Tables 1 and 2). In the MP41 xenograft, a tumor growth delay was observed in the 3 groups of combined treatments, i.e. concomitant fotemustine/S44563, fotemustine first then S44563 since day 43, and concomitant fotemustine/S44563 then S44563 since day 43 (data not shown). The median OS was 44 days for the control group, 67 days for the S44563 group, 72 days for the fotemustine group, and 102 days for the fotemustine+S44563 (whatever the schedule of combination) (p<10^−3^) (Figure 4B). In the MP77 xenograft, concomitant S44563 (100 mg/kg per injection) and fotemustine (15 mg/kg) induced a more significant TGI at day 35 after start of treatment than fotemustine alone (p = 0.04) (Figure 4C). Moreover, an increased median overall survival was observed when S44563 was administered in an adjuvant setting after fotemustine-induced complete remission. In the MM26 xenograft, concomitant S44563 (50 mg/kg per injection) and fotemustine (30 mg/kg) slightly improved the TGI in comparison to fotemustine alone, but significantly improved the complete remission rate (p<0.02) (Table 2). In contrast, because of the occurrence of toxic deaths when S44563 was administered after fotemustine since day 43, we have not observed prolonged survival. This toxicity was observed in the only one MM26 model and could not be clearly explained due to its restrictive occurrence in the MM26 UM PDX. Finally, in the MM66 xenograft, concomitant S44563 (50 mg/kg per injection) and fotemustine (30 mg/kg) significantly enhances efficacy compared to treatment with either agent alone (p<0.003) (Figure 4D).

**Table 2:** Overall and median survival after combination of S44563 and fotemustine.

Models	Criteria	Control	S-50	F-30	F-30+S-50	F-30/S-100	F-30+S-50/S-100
**MP41**	**OS (78)**	8%	14%	21%	77%	77%	69%
	**Median S.**	44	61	72*	102*		
**MP77**	**OS (85)**	0%	0%	0%	12%	48%	67%
	**Median S.**	7	11	67	59	82	>85
	**Prolonged CR (85)**	/	/	20%	20%	40%○	60%○
**MM26**	**OS (117)**	0%	0%	90%	89%	/	/
	**CR rate**	0%	0%	0%	29%		

**Abbreviations:** F-30/S-50, fotemustine followed by S44563 adminstration since day 43; OS (day), overall survival observed at the indicated day; Median S, median survival in days since start of treatment.

F-30 versus F-30+S-50 or F-30/S-100: p = 0.04.

F-30/S-100 versus F-30+S-50/S-100: p = 0.038.

### Correlations between IHC expressions and ORR to S44563

We then evaluated correlations between histopathological analyses and response to S44563. We first showed that high Bcl-2 expressing tumors could be either sensitive or resistant to S44563 administered alone (Figure 2 and Table 1). A similar observation could be done for high Mcl-1 expressing tumors. In contrast, only MP41 xenograft with a high Bcl-X_L_ expression was sensitive to S44563, whereas the 3 other models characterized by a low Bcl-X_L_ expression were resistant to S44563 administered alone. However, because of the limited number of tested xenografts, it was not possible to determine the relation between the global pathological score, defined as: (Bcl-2 score)+(Bcl-X_L_ score)/Mcl-1 score, and responses to S44563.

### Immunohistochemical studies after S44563 administration

**Figure 5:**
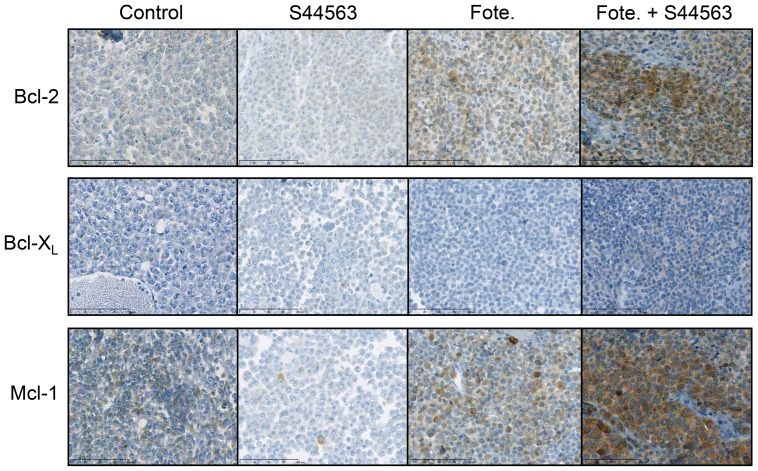
Immunohistochemical analyses under S44563 administration. Immunohistochemical analyses of the MM66 UM xenograft after S44563, fotemustine, or S44563+fotemustine.

In order to evaluate the impact of S44563 on the expression of Bcl-2, Bcl-X_L_, and Mcl-1 proteins, two to four tumor samples have been collected at the time of mice sacrifice for immunohistochemical studies. IHC analyses showed that Bcl-2, Bcl-X_L_, and Mcl-1 expressions were not modified after S44563 administration (Table S3) (Figure S4). Surprinsingly, we have observed that fotemustine alone induced a dramatic increase of Bcl-2 in the MM66 xenograft, and an increase of the Mcl-1 protein in the MP41 and MM66 xenografts. Consequently, the expression of both Bcl-2 and Mcl-1 was highly increased after concomitant administration of fotemustine and S44563 in the MM66 xenograft (Figure 5).

In order to evaluate *in vivo* apoptosis induction, we have studied cleaved caspase-3 expression before and after S44563 +/− fotemustine administration. Tumor samples have also been collected at the time of mice sacrifice. We did not detect significant increase of activated caspase-3 in the four treated UM xenografts (data not shown). Similarly, we did not observe LC3-positive tumor cells before and after treatments (data not shown), as in *in vitro* experiments, except in one MM66 tumor among five xenografts treated by S44563 (50 mg), where a unique islet of about 20 positive cells has been observed.

## Discussion

Using four well-characterized uveal melanoma PDXs, we have shown that a specific Bcl-2/Bcl-X_L_ inhibitor only had a moderate efficacy when administered alone, except in one model (MP41) with a clear dose-dependent *in vivo* efficacy. However, we have shown that this Bcl-2/Bcl-X_L_ inhibition significantly increased the antitumor activity of fotemustine in these preclinical models. Indeed, in 3 out of the 4 tested xenografts (MP41, MP77, and MM66), S44563 increased tumor growth inhibition when combined to fotemustine in comparison to fotemustine alone. It also increased the complete remission rate (MM26 model) and the overall survival (MP41 model). Overall, these data strongly suggest that targeting Bcl-2/Bcl-X_L_ in combination with fotemustine significantly improves, in human UM xenografts, response to the alkylating agent administered alone.

Hence, our treatment combination significantly enhanced efficacy compared to treatment with either agent alone, confirming positive data reported with the BH3-mimetic ABT-737 and various chemotherapies such as fotemustine [31], temozolomide [31], [32], melphalan [33], cytosine arabinoside [34], gemcitabine [35], actinomycine D [36], carboplatin [37], and others, or with Bcl-2 antisense and cyclophosphamide [38] or chlorambucil [39]. In the MM66 xenograft, this observation could be explained by the fact that fotemustine highly increased Bcl-2 expression that could be therefore more efficiently inhibited. In few reports, it has been shown that this effect was p53-independent but related to the pro-apoptotic protein Noxa [31], [32], [40], which inactivates other Bcl-2-family members such as Mcl-1 or A1 proteins [40]. The balance of Bcl-2/Mcl-1 proteins has also been largely shown to impact the efficacy of ABT-737 [34]–[36], [41], [42], suggesting that modulation of Noxa and Mcl-1 could be used as a strategy for sensitizing tumor cells to ABT-737 [31], [43]. Another predictive marker could be Bcl-X_L_ expression and indeed in our *in vivo* experiments, the best responding xenografts were characterized by higher Bcl-X_L_ expression, suggesting that specifically targeting the Bcl-X_L_ protein may be an efficient therapeutic approach by itself. Such an observation is confirmed by the report of Jain and Meyer-Hermann who showed that inhibition of Bcl-X_L_ may decrease the reparation of DNA damages induced by carboplatin [37].

One other mechanism leading to the synergistic effect between a Bcl-2 inhibitor and temozolomide could be the specific induction of autophagic cell death, as recently reported [44], [45]. In our *in vitro* experiments testing the effect of S44563 in 3 human UM cell lines, S44563 did not modify the proportion and the total amount of cleaved LC3 protein. Whereas we have not confirmed the modulation of autophagy, further *in vivo* experiments are needed to more extensively study the impact of such Bcl-2/Bcl-X_L_ inhibitor in human uveal melanoma tumors.

Taking together, our results clearly demonstrate that Bcl-2/Bcl-X_L_ targeting is a promising therapeutic approach for uveal melanoma. Despite a modest efficacy when used alone, S44563 significantly increased efficacy of fotemustine. Even intra-arterial hepatic administration of this alkylating agent remains poorly efficient in UM patients, both in an adjuvant setting after proton beam irradiation [46] and in metastatic evolution [47]. It is therefore required to improve its efficacy and reverse mechanisms of resistance. Targeting specific members of the Bcl-2 family, such as Bcl-2 and Bcl-X_L_ proteins, could be of high clinical interest. In our *in vivo* experiments, we have evaluated this targeted approach in relevant preclinical models and showed that both in concomitant and differed administration, S44563 were able to increase the effect of fotemustine. Our data therefore suggest that S44563 could probably be evaluated in both adjuvant and metastatic settings of UM patients. In the first clinical situation, genomic features of UM tumors clearly defined prognostic outcome of the patients [48], [49], allowing a relevant discrimination and selection of poor prognosis cases, and, in the second clinical situation, the overall prognosis remains very poor. Altogether, our results emphasize a potential therapeutic interest for targeting Bcl-2/Bcl-X_L_ UM patients.

## Supporting Information

Figure S1 Chemical structure of S44563.
(TIF)

Figure S2 Viability of the 3 uveal melanoma xenograft-derived cell lines MP41, MM26, and MM66 after 24 hours incubation with S44563. Viability of treated cells was determined by the 50% inhibitory concentration induced by S44563 using a WST-1 test. A two-way ANOVA with Bonferroni post-test was then performed (* means a p<0.05).
(TIF)

Figure S3 Overall response rate (ORR) after S44563 administration. A. ORR of mice treated by S44563 at a dose of 50 mg/kg per injection. B. ORR of mice treated by S44563 at a dose of 100 mg/kg per injection. The ORR was defined as the relative tumor volume variation (RTVV) of each S44563-treated mouse calculated from the following formula: [(Vt/Vc)−1], where Vt is the volume of the treated mouse and Vc the median volume of the corresponding control group at a time corresponding to the end of treatment.
(TIF)

Figure S4 Immunohistochemical analyses under S44563 administration. A. Bcl-2 expression determined by immunohistochemical analyses of the MM66 xenograft after S44563 *in vivo* administration. **B.** Determination of Bcl-2-positive tumor cells in the 4 UM xenografts after S44563 administration. **C.** Determination of Bcl-X_L_-positive tumor cells in the 4 UM PDXs after S44563 administration. **D.** Determination of Mcl-1-positive tumor cells in the 4 UM xenografts after S44563 administration.
(TIF)

Table S1 Biological characteristics of the 4 UM PDXs.
(DOC)

Table S2 Immunohistochemical analyses of the 4 UM PDXs.
(DOC)

Table S3 IHC scores under S44563 +/− fotemustine administration.
(DOC)
