# Supplementary figures and images for: Targeting Bcl-2/Bcl-XL Induces Antitumor Activity in Uveal Melanoma Patient-Derived Xenografts
Source: PLoS One. 2014 Jan 13;9(1):e80836. doi: 10.1371/journal.pone.0080836 (PMC3890263; doi:10.1371/journal.pone.0080836)

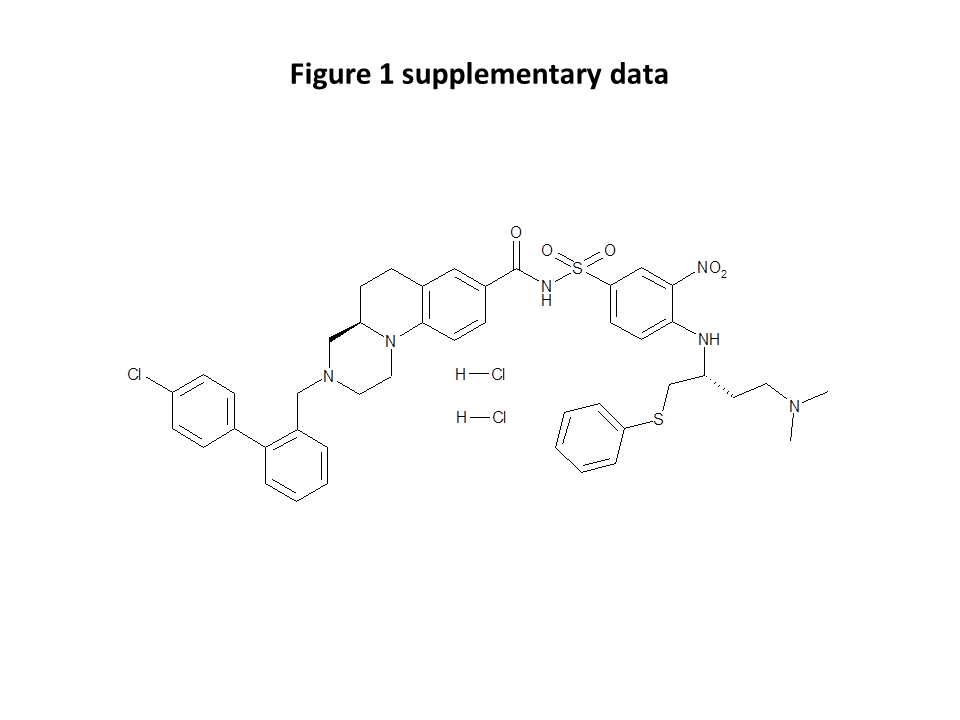

Supplement: Figure S1 — Chemical structure of S44563. (TIF) [file pone.0080836.s001.tif]

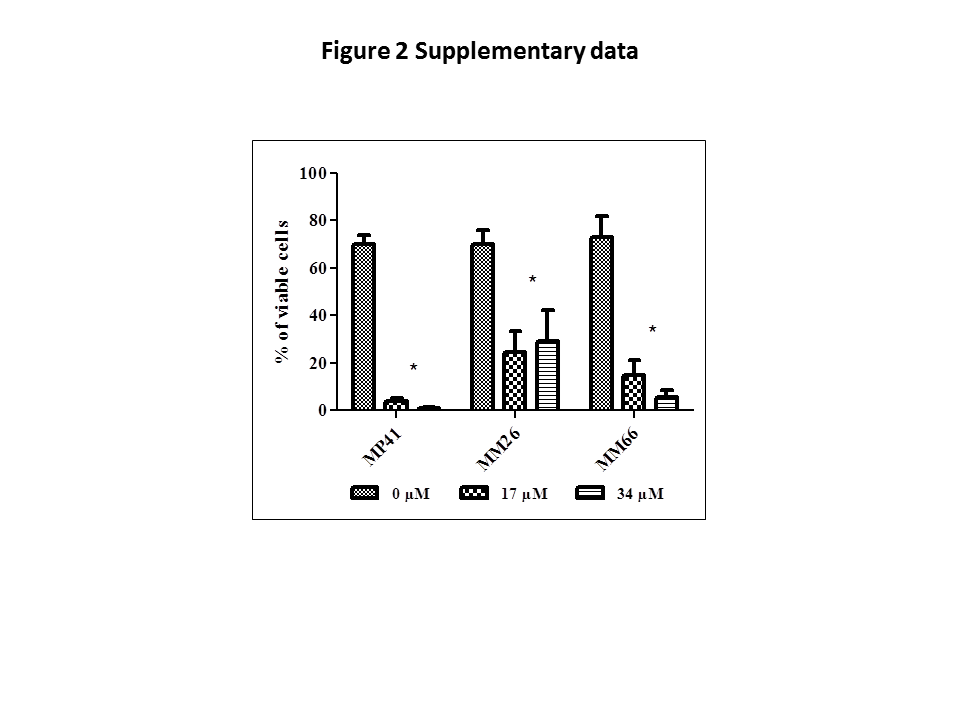

Supplement: Figure S2 — Viability of the 3 uveal melanoma xenograft-derived cell lines MP41, MM26, and MM66 after 24 hours incubation with S44563. Viability of treated cells was determined by the 50% inhibitory concentration induced by S44563 using a WST-1 test. A two-way ANOVA with Bonferroni post-test was then performed (* means a p<0.05). (TIF) [file pone.0080836.s002.tif]

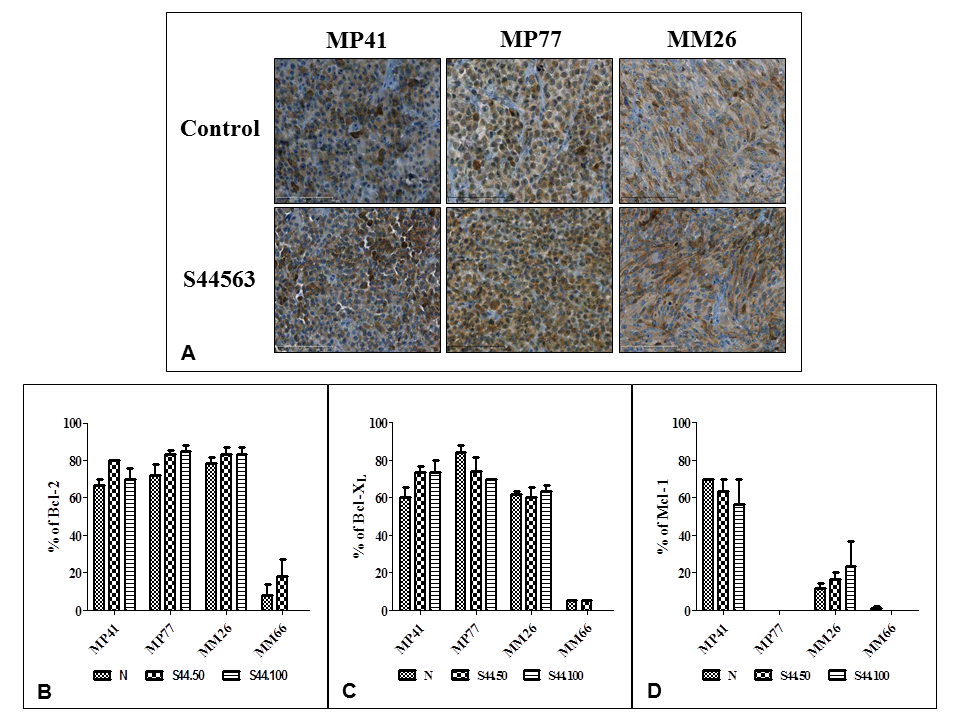

Supplement: Figure S4 — Immunohistochemical analyses under S44563 administration. A. Bcl-2 expression determined by immunohistochemical analyses of the MM66 xenograft after S44563 in vivo administration. B. Determination of Bcl-2-positive tumor cells in the 4 UM xenografts after S44563 administration. C. Determination of Bcl-XL-positive tumor cells in the 4 UM PDXs after S44563 administration. D. Determination of Mcl-1-positive tumor cells in the 4 UM xenografts after S44563 administration. (TIF) [file pone.0080836.s004.tif]
