# Supplementary material for: Targeting Bcl-2/Bcl-XL Induces Antitumor Activity in Uveal Melanoma Patient-Derived Xenografts
Source: PLoS One. 2014 Jan 13;9(1):e80836. doi: 10.1371/journal.pone.0080836 (PMC3890263; doi:10.1371/journal.pone.0080836)
Supplement: Table S1 — Biological characteristics of the 4 UM PDXs. (DOC) [file pone.0080836.s005.doc]

**Table 1S: Biological characteristics of the 4 UM PDXs**

| **Xenografts** | **Histology** | **GNAQ** | **GNA11** | **BAP1** | **BRAF** | **L3** |
| --- | --- | --- | --- | --- | --- | --- |
| **MP41** | Epithelioid | 0 | + | 0 | 0 | 0 |
| **MP77** | Epithelioid | 0 | + | 0 | 0 | + |
| **MM26** | Epithelioid | + | 0 | 0 | 0 | + |
| **MM66** | Epithelioid | 0 | + | 0 | 0 | 0 |
