# Supplementary material for: Targeting Bcl-2/Bcl-XL Induces Antitumor Activity in Uveal Melanoma Patient-Derived Xenografts
Source: PLoS One. 2014 Jan 13;9(1):e80836. doi: 10.1371/journal.pone.0080836 (PMC3890263; doi:10.1371/journal.pone.0080836)
Supplement: Table S2 — Immunohistochemical analyses of the 4 UM PDXs. (DOC) [file pone.0080836.s006.doc]

**Table 2S: Immunohistochemical analyses of the 4 UM PDXs**

| **Models**  **(n° tumors)** | | **Bcl-2** | | | **Bcl-X_L_** | | | **Mcl-1** | | | **Global**  **score** |
| --- | --- | --- | --- | --- | --- | --- | --- | --- | --- | --- | --- |
|  |  | **%** | **I** | **H-score** | **%** | **I** | **H-score** | **%** | **I** | **H-score** |  |
| **MP41** | 252 | 60 | 2/3 | 150 | 70 | 2/3 | 175 | 50 | 2 | 100 | 3.25 |
|  | 253 | 70 | 2/3 | 175 | 70 | 2/3 | 175 | 60 | 1/2 | 90 | 3.89 |
|  | 254 | 70 | 2 | 140 | 70 | 2/3 | 175 | 70 | 1/2 | 105 | 3 |
| **MP77** | 709 | 80 | 2 | 160 | 0 | 0 | 0 | 80 | 1/2 | 135 | 1.19 |
|  | 710 | 50 | 2 | 100 | 0 | 0 | 0 | 90 | 1/2 | 135 | 0.74 |
|  | 711 | 80 | 2 | 160 | 0 | 0 | 0 | 90 | 1/2 | 135 | 1.19 |
|  | 712 | 80 | 2 | 160 | 0 | 0 | 0 | 90 | 1/2 | 135 | 1.19 |
|  | 713 | 70 | 2 | 140 | 0 | 0 | 0 | 70 | 1/2 | 135 | 1.04 |
| **MM26** | 953 | 80 | 2/3 | 200 | 5 | 1/2 | 7,5 | 60 | 1/2 | 90 | 2.31 |
|  | 954 | 70 | 2/3 | 175 | 5 | 1 | 5 | 60 | 1/2 | 90 | 2 |
|  | 955 | 70 | 2/3 | 175 | 10 | 1 | 10 | 60 | 1/2 | 90 | 2.06 |
| **MM66** | 020B | 0 | 0 | 0 | 0 | 0 | 0 | 5 | 1/2 | 7,5 | 0 |
|  | 020C | 0 | 0 | 0 | 0 | 0 | 0 | 5 | 1/2 | 7,5 | 0 |
|  | 020L | 5 | 1 | 5 | 0 | 0 | 0 | 5 | 1/2 | 7,5 | 0.67 |
|  | 020M | 5 | 1 | 5 | 0 | 0 | 0 | 5 | 1/2 | 7,5 | 0.67 |

**Abbreviations:** %, % of positive tumor cells; I, staining intensity; H, IHC score defined as:

% x I.
