# Supplementary material for: Targeting Bcl-2/Bcl-XL Induces Antitumor Activity in Uveal Melanoma Patient-Derived Xenografts
Source: PLoS One. 2014 Jan 13;9(1):e80836. doi: 10.1371/journal.pone.0080836 (PMC3890263; doi:10.1371/journal.pone.0080836)
Supplement: Table S3 — IHC scores under S44563 +/− fotemustine administration. (DOC) [file pone.0080836.s007.doc]

**Table 3S: IHC scores under S44563 +/- fotemustine administration**

| **Proteins** | **Treatments** | **MP41** | **MP77** | **MM26** | **MM66** |
| --- | --- | --- | --- | --- | --- |
| **Bcl-2** | **Control** | 150 | 160 | 200 | 5 |
|  | **S44563** | 200 | 170 | 200 | 15 |
|  | **Fotemustine** | 150 | / | 200 | 75 |
|  | **S + F** | 175 | / | 225 | 115 |
| **Bcl-X_L_** | **Control** | 175 | 0 | 20 | 5 |
|  | **S44563** | 175 | 0 | 22 | 0 |
|  | **Fotemustine** | 75 | / | 10 | 0 |
|  | **S + F** | 75 | / | 20 | 0 |
| **Mcl-1** | **Control** | 100 | 180 | 120 | 8 |
|  | **S44563** | 130 | 140 | 135 | 12 |
|  | **Fotemustine** | 175 | / | 120 | 125 |
|  | **S + F** | 90 | / | 140 | 138 |

**Abbreviations:** S, S44563; F, fotemustine.
